# Solving the second-order free rider problem in a public goods game: An experiment using a leader support system

**DOI:** 10.1038/srep38349

**Published:** 2016-12-09

**Authors:** Hiroki Ozono, Nobuhito Jin, Motoki Watabe, Kazumi Shimizu

**Affiliations:** 1 Faculty of Law, Economics and Humanities, Kagoshima University, 1-21-30, Korimoto, Kagoshima 890-0065, Japan; 2 School of Psychology Practices, College of Integrated Human and Social Welfare Studies, Shukutoku University, 200, Daiganji-cho, Chuo-ku, Chiba 260-8701, Japan; 3 School of Business, MonashUniversity, Malaysia, Jalan Lagoon Selatan, 46150 Bandar Sunway, Selangor Darul Ehsan, Malaysia; 4 School of Political Science and Economics, Waseda University, Nishi-Waseda, Shinjuku-ku, Tokyo 169-8050, Japan

## Abstract

Punishment of non-cooperators—free riders—can lead to high cooperation in public goods games (PGG). However, second-order free riders, who do not pay punishment costs, reduce the effectiveness of punishment. Here we introduce a “leader support system,” in which one group leader can freely punish group followers using capital pooled through the support of group followers. In our experiment, participants engage in three stages repeatedly: a PGG stage in which followers decide to cooperate for their group; a support stage in which followers decide whether to support the leader; and a punishment stage in which the leader can punish any follower. We compare a support-present condition with a no-support condition, in which there is an external source for the leader’s punishment. The results show that punishment occurs more frequently in the support-present condition than the no-support condition. Within the former, both higher cooperation and higher support for a leader are achieved under linkage-type leaders—who punish both non-cooperators and non-supporters. In addition, linkage-type leaders themselves earn higher profits than other leader types because they withdraw more support. This means that leaders who effectively punish followers could increase their own benefits and the second-order free rider problem would be solved.

The difficulties of constructing a cooperative relationship are formulized as a public goods problem^1^,^2^, and many such studies are conducted in the social sciences. In a typical public goods game (PGG), group members decide how much of their own resources to contribute to the common pool and the resources gathered in the pool benefit members equally. The entire group earns the highest profit when all members contribute all their resources. Free riders, however, can increase their own payoff if they contribute nothing and still benefit from the common pool. This results in a socially inefficient situation. This is called the free rider problem. Humans encounter many PGG situations in daily life. In historical times, PGG situations involved food distribution in hunter–gatherer societies and irrigation facility work in agrarian societies. In modern times, such PGG situations range widely from small-scale issues, like housework distribution within a household, to large-scale ones, like acts to prevent global warming.

Peer punishment is one solution proposed by scholars^3^,^4^,^5^,^6^. This involves individuals punishing free riders, which decreases the incentive to free ride and, thus, establishes cooperation^7^. Despite several laboratory experiments indicating that peer punishment solves the free rider problem^3^,^4^,^5^,^6^, several theoretical and empirical questions have been posed. The biggest theoretical issue is the second-order free rider problem^8^,^9^. Because punishing someone incurs cost, owing the punishment cost to maintain group cooperation is a second-order cooperative action. However, because the individual’s profits increase if s/he punishes nobody, second-order free riders emerge. Thus, in theory, peer punishment should not evolve. The reasons people punish, even if they have to pay the cost, have been proposed using multi-level selection theory^10^ and reputational benefits for punishers^11^,^12^. However, both theories have been criticized (Pinker’s criticism^13^ for multi-level selection; Raihani and Bshary’s criticism^14^ for punishers’ reputations) and there is no sufficient answer yet. Furthermore, an anthropological survey showed that punishment between individuals is rare in a small society, which is similar to an evolved environment^15^. As such, there is much doubt as to whether peer punishment solely can solve the public goods problem.

Other than the peer punishment system, the pool punishment system has been proposed to solve the public goods problem^16^,^17^,^18^,^19^,^20^. Sigmund *et al*.^18^ compares peer punishment with pool punishment, in which group members pay costs to a punishment-executing system (e.g., a police force) and the system uses these resources as capital to punish free riders. The authors mathematically show that the pool punishment system is more stable than peer punishment only when the system punishes both the first- and second-order free riders (the latter do not bear the cost of the punishment system). Traulsen *et al*.^20^ examine the pool punishment system by implementing it in a laboratory experiment and show that participants tend to select pool punishment over peer punishment. In addition, the authors report that systems with second-order punishment increase the number of people bearing the punishment cost, and thus, high cooperation is likely compared to the condition with only first-order punishment.

In our study, we postulate the executor of pool punishment as a leader. In previous studies on pool punishment, the executor of punishment is assumed to be a system governed by all group members and punishment is executed automatically in accordance with its rules. Thus, the profit of the punishment system itself is not considered. Indeed, these systems have existed in actual societies^21^. In many cases, however, each pool system is governed by a leader or a few leaders, such as headmen in villages, lords of manors, or kings in nations. In such systems, followers support their leaders by giving their own resources to their leaders. We call this the “leader support system”, whereby a leader can freely decide the punishment rules: whom to punish to what degree. In addition, it should be considered that the leader could obtain surplus resources that were pooled by followers but not used as the punishment cost. In previous studies on pool punishment, no one can obtain surplus resources because they should be used to maintain the pool punishment system even if all members were cooperators^18^,^19^,^20^. Although maintenance cost is necessary, we consider that surplus resources should remain if enough resources are pooled to the leader, giving the leader incentive to induce support from followers. Under the leader support system, a leader can obtain more support and profit by executing both the first- and second-order punishment, which also results in high cooperation in PGG. Considering the group leader as a punishment executor makes it possible to reveal the origin of the punishment rule: how and why did the second-order punishment emerge?

Our study aims to address the issues within the pool punishment system by setting up a leader support system. Under this system, a specific person, namely, a group leader, can freely punish group followers using resources pooled through support from group followers as capital. Specifically, in a group experiment conducted in a laboratory, one participant is assigned as the leader and other participants as group followers. The participants are asked to engage in three stages: a PGG stage in which all the followers engage in PGG; a support stage in which the followers have opportunity to provide support for the leader; and a punishment stage in which the leader can freely punish followers. Our study examines the behavior of the leader and followers and the group cooperation level in a PGG.

When a leader support system is postulated, first, the leader might have an incentive to punish those who do not support him/her (termed “self-focused punishment”) to induce support. Under such a self-focused punishment leader, non-cooperators in PGG are not punished and high cooperation is not attained. As a result, a tyrannical state might arise in which the followers continue to support the leader for fear of punishment. In other words, under a self-focused punishment leader, a distorted state might arise in which second-order free riders would be punished but first-order free riders would not.

Second, under a leader support system, the leader might have another incentive to punish non-cooperators in the PGG (termed “group-focused punishment”) in order to gain the trust of the followers as an effective leader and induce support. Under such a group-focused punishment leader, non-supporters are not punished and resources for punishment might eventually be insufficient. This would lead to difficulty achieving high PGG cooperation. This means that the second-order free riders would increase as only the first-order free riders are punished, resulting in difficulty maintaining high PGG cooperation.

What happens under a “linkage punishment” leader—who executes both self-focused and group-focused punishment? Such a linkage punishment leader would be supported because s/he punishes the second-order free riders, and s/he would achieve a high cooperation in the PGG because s/he punishes the first-order free riders by using gathered support as capital. In addition, linkage punishment leaders would be more likely to gain support from followers compared to self-focused punishment leaders because the former are beneficial to the group and gain followers’ trust. Thus, the leader’s own profit is expected to increase.

Under a peer punishment system, individuals have no incentives to bear the cost for punishing the first- and second-order free riders. Under a leader support system, on the other hand, leaders have incentives to bear both the first- and second-order punishment costs in order to attain support from followers. Furthermore, followers have incentive to bear the punishment cost (i.e., to support the leader) in order to evade punishment from the leader. Therefore, the second-order free rider problem can be solved under the leader support system if a linkage punishment leader appears. This creates a state in which both the followers and leader can earn profits. This idea of solving the second-order free rider problem under a linkage punishment leader is originally proposed in evolutionary simulation research by Matsumoto and Jin^22^. Through a laboratory experiment, we attempt to demonstrate for the first time that a linkage punishment leader actually appears under the leader support system, which makes it easier to achieve high cooperation in the PGG.

There are a few laboratory experiments in which only one leader can punish others in PGG^23^,^24^. While these are pioneering studies on leader punishment, they are critically different from the leader support system in our experiment. One problem in these previous studies is that the capital for punishment is provided externally, not from the support by followers. When leaders have no chance to obtain support from followers, leaders cannot obtain any benefit by punishing followers, resulting in significant decrease of the incentive for punishment. By contrast, the leader support system we introduce provides an incentive for leaders to punish the first- and second-order free riders by receiving support from followers. By comparing conditions with and without support, our study attempts to clarify the function of support by followers in leader punishment. We predict that punishment by leaders would be heavier and high cooperation would be more likely to be achieved in the leader punishment with support from followers than without support.

In our experiment, six-person groups are assembled, comprising five followers who engage in a PGG and one leader executing punishment. Thereafter, settings include whether capital for the leader’s punishment come from followers’ support (support-present condition) or from the outside (no-support condition). There are three stages in the experiment. In the first stage (PGG), each of the six members, including the leader, is given 100 tokens and five followers decide whether to contribute all 100 tokens to the group pool. The sum of the contributed tokens is doubled and distributed equally to five followers except for the leader. In the second stage (support), an additional 20 tokens are provided to each of the six members in the support-present condition. The five followers other than the leader decide whether to provide (support) the 20 tokens to the leader. If a follower decides to support the leader, the leader obtains the 20 tokens. In the no-support condition, each member is given tokens, but there is nothing for any group follower or the leader to decide in this condition, that is, they simply receive the tokens. In the third stage (punishment), the leader uses the amount earned in the second stage as capital for punishment, and the leader determines, in increments of 20 tokens, how much to reduce each follower’s tokens. The punishment rate is double, meaning that if a leader uses 20 tokens to punish a certain follower, the follower would lose 40 tokens. The amount the leader does not use for punishment is added to the leader’s own profit. These three stages are repeated 15 times.

Four hypotheses are set based on the abovementioned arguments.

1. H1: Punishment toward non-cooperators in the PGG is more likely to occur in a support-present condition than a no-support condition (H1a), making it likely that high cooperation is achieved (H1b).
2. H2: Within a support-present condition, the linkage punishment leader executes stronger punishment to PGG non-cooperators than the non-linkage punishment leaders do (H2a), making it easier to achieve high PGG cooperation (H2b).
3. H3: Within a support-present condition, the linkage punishment leader executes stronger punishment for non-supportive followers than the non-linkage punishment leaders do (H3a), making it easier to achieve a high level of support (H3b).
4. H4: From H2 and H3, the profits of the linkage punishment leaders are higher than those of the non-linkage punishment leaders (H4a) and the profits of the followers are higher under the linkage punishment leader than under the non-linkage punishment leader (H4b).

## Results

### Support-present condition versus no-support condition

First, the support-present condition and no-support condition are compared. We use the average total contribution of the five followers in the PGG through all 15 periods as the index of cooperation level and the leaders’ average punitive amount for each non-contributor as a punishment index. We do not use a simple average punitive amount as the punishment index because the number of non-contributors, who are usually the target of punishment, greatly varies depending on the group and period. Thus, the simple punitive amount varies according to the number of non-contributors as well. To control the influence of the number of non-contributors, the average punitive amount for each non-contributor is calculated for each group as follows: the total punitive amount to non-contributors for the 15 periods is divided by the total number of non-contributors for the 15 periods. The calculated amounts are used as the punishment index.

**Figure 1:**
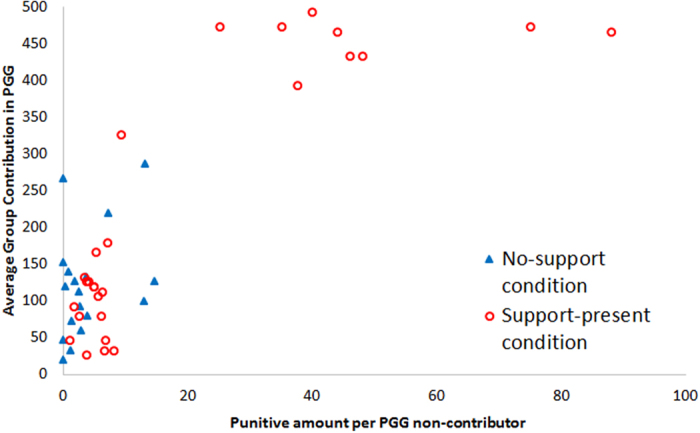
Correlation between average punitive amount per PGG non-contributor and average group contribution in the PGG for the 15 periods. The 18 triangle markers are the data for the no-support condition and the 27 circle markers are the data for the support-present condition.

Figure 1 indicates the correlation between the average contributions in the PGG and the average punitive amount for each non-contributor. The results indicate the leaders in the support-present condition punished more non-contributors than the leaders in the no-support condition did (Mann–Whitney U-test: *p* = 0.001). As for the average group contribution in the PGG, there is no significant difference between the conditions (Mann–Whitney U-test: *p* = 0.144). However, using the definition of “high cooperation level” as an average contribution of more than 80%, 8 out of 27 groups (29.6%) in the support-present condition and none of 18 groups (0%) in the no-support condition achieve a high cooperation level, which is statistically significant (Fisher’s exact test: *p* = 0.014). By setting the definition of “high cooperation level” to more than 70% and 60%, similar statistical significance is observed (*p* = 0.007 and *p* = 0.003, respectively). Thus, high PGG cooperation is achieved more easily for the support-present condition. This is not statistically significant with the U-test because polarization of high and low cooperation occurred in the support-present condition. Furthermore, there is a strong positive correlation between average punitive amount per non-contributor and average total PGG contribution (*r* = 0.83, *p* < 0.001). These results support H1a and H1b.

### Comparing punishment types by leaders in the support-present condition

Within the support-present condition, we analyze what type of punishment achieves higher cooperation. First, we calculate the percentage of punishment that the leaders impose on each of the four follower’s behavior—“followers who contribute in PGG and support the leader”, “followers who do not contribute in PGG but support the leader”, “followers who contribute in PGG but do not support the leader”, and “followers who do not contribute in PGG and do not support the leader” (see Supplementary Table S1 for the data of percentages for each group) through all 15 periods. Second, we exclude the data of the two groups in which the leaders punish the type of followers who contribute in PGG and support the leader, because it is difficult to interpret the meaning of and motivation for this type of punishment. We confirm that the same results are obtained even when we include the data of these two groups (see Supplementary analysis 1).

Then, we categorize leaders’ punishment patterns. Linkage punishment (L) refers to a leader who punishes all the other three followers’ behavioral patterns at least once, which means the leaders punish both non-contributors and non-supporters, and the other patterns are categorized as leaders of the non-linkage (NL) type. There are 10 L-type and 15 NL-type leaders. For the 15 periods, we calculate the average total PGG contribution, average total support amount, average punitive amount per non-contributor, average punitive amount per non-supporter, leader’s average profit, and followers’ average profit per group. Thereafter, a Mann–Whitney U-test is conducted to determine whether a difference exists between L-type and NL-type leaders. The results show that there is a significant difference in all categories, implying that the following hypotheses are supported: H2a and H2b (i.e., under L-type leaders, punishment toward a non-cooperator is more strongly imposed (*p* < 0.001), and thus, PGG cooperativeness is higher (*p* < 0.001)); H3a and H3b (under L-type leaders, punishment toward non-supporters is more strongly imposed (*p* = 0.002), and therefore, support toward the leader is higher (*p* < 0.001)); and H4a and H4b (as a result of these developments, profit of the L-type leader is higher (*p* = 0.001) and followers’ profits under the L-type leader are higher (*p* < 0.001)).

Next, punishment types are categorized more specifically. We categorize 15 NL-type leaders into two more specific categories. Group-focused punishment (G) refers to leaders who punish only non-contributors, that is, punish only followers who do not contribute but support the leader and followers who do not contribute and do not support the leader, while never punishing followers who contribute and do not support the leader. Self-focused punishment (S) refers to leaders who punish only non-supporters, that is, punished followers who contribute and do not support the leader and followers who do not contribute and do not support the leader, while never punishing followers who do not contribute but support the leader. There are five S-type, and seven G-type leaders. Three leaders do not fit any of these L, S, and G categories. These three leaders punish only followers who do not contribute and do not support the leader. They are few in number and cannot be characterized as L, S, or G type. Thus, they are excluded from the analysis (see Supplementary Table S1 for detailed information about leader categorization).

**Figure 2:**
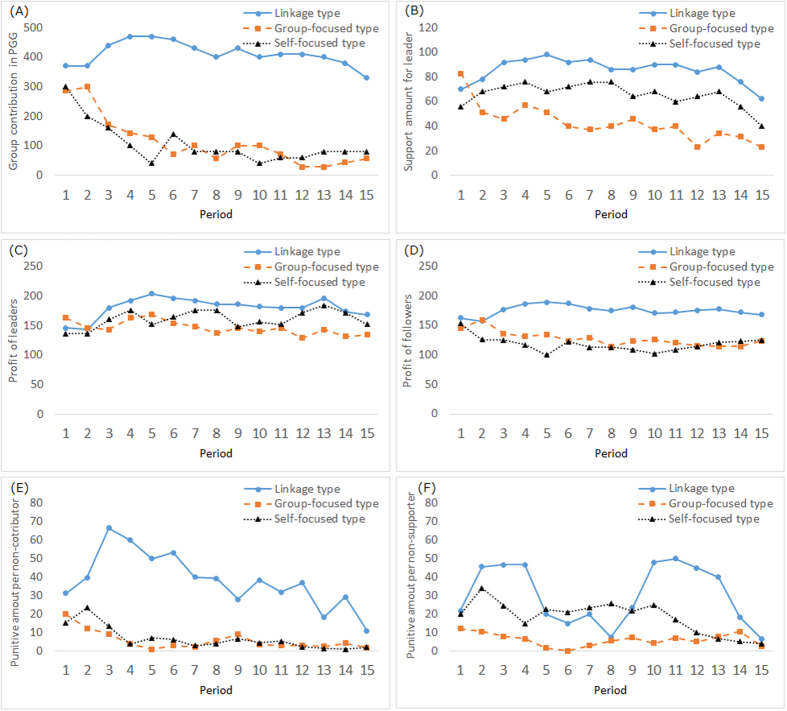
Average group contribution to the public good (**A**), average support amount for the leader (**B**), average punishment for one non-contributor in the PGG (**C**), average punishment for one non-supporter (**D**), average profit of leaders (**E**), and average profit of followers (**F**) over 15 periods of play under the linkage leader (N = 10), group-focused leader (N = 7), and self-focused leader (N = 5).

The total PGG contribution, total support amount to the leader, punitive amount per non-contributor, punitive amount per non-supporter, and average profit of leader and followers are calculated for each period (see Fig. 2). The punitive amount per non-contributor and punitive amount per non-supporter are impossible to calculate in some periods because there are no non-contributors or non-supporters in those periods. We exclude these data when calculating the averages.

The Mann–Whitney U-test is conducted to determine whether a difference exists between the leader types. We use the average indexes for the 15 periods. Bonfirroni’s correction is used to determine the significance of comparisons of the three leader types—L, S, and G—from this point onward. As for the total PGG contribution, the followers under L-type leaders contribute more to the PGG pool than under S-type leaders (*p* = 0.013) and G-type leaders (*p* = 0.002). The total support for L-type leaders is higher than that for S-type leaders (*p* = 0.024) and G-type leaders (*p* < 0.001).

The average punitive amount per non-contributor is larger for L-type leaders than for S-type (*p* = 0.004) and G-type leaders (*p* < 0.001). Concerning the average punitive amount per non-supporter, L-type leaders punish more than G-type leaders do (*p* = 0.004), while there is no difference between L- and S-type leaders (*p* = 0.388).

Finally, the average profit of leaders and followers is analyzed. The Mann–Whitney U-test reveals that the leader’s profit is higher for L-type leaders than for G-type leaders (*p* = 0.004) because G-type leaders cannot attain support and their profit remains low. There is no statistically significant difference between L- and S-type leaders (*p* = 0.315). This shows that S-type leaders reach a certain profit amount by attaining support. As for the followers, the Mann–Whitney U-test reveals that followers under L-type leaders have higher profits than those under S-type leaders (*p* = 0.008) and those under G-type leaders (*p* = 0.009), which results from achieving higher PGG cooperation under L-type leaders than under S-type or G-type leaders (for further analyses using another categorization of leaders or non-categorization of leaders, see Supplementary analysis 2). In addition, to examine the superiority of the leader within a group, the difference in average profit between leaders and followers is calculated (L types: 5.2; S types: 42.8; G types: 18.8) and analyzed using a U-test. The results show that S-type leaders have higher profit differences than L-type (*p* = 0.003) and G-type leaders (*p* = 0.049) do, which means that the S-type leaders are likely to hold relatively dominant positions within the group.

## Discussion

In our experiment, we compare the no-support condition with the support-present condition. We show that no-support condition groups are less likely to achieve high cooperation in PGG than support-present condition groups, because punishment to non-contributors is weaker in the no-support condition. These results are understandable because the leader has no incentive to punish in the no-support condition. By contrast, Baldassarri *et al*.^23^ show that high cooperation is achieved under a leader in spite of no support by followers. At first glance, this might seem contradictory to our results. However, the participants in Baldassarri *et al*.^23^ include acquaintances, and thus, punishment might have been executed in consideration of future reputation (23, p. 11026). In a completely anonymous situation, in which no reputation spreads, like our experiment, it is difficult to solve the public goods problem by a leader without the support of his or her followers.

In our analysis, we mainly focus on how the leader’s punishment type affects the behavior and profits of both followers and a leader within a support-present condition. The results show that an L-type leader strongly punishes PGG non-cooperators—the first-order free riders—making it easier to achieve high cooperation. Furthermore, L-type leaders strongly punish non-supporters—the second-order free riders—making a high support level easy for the leader to maintain punishment. In addition, compared to other punishment types, both L-type leaders and their followers earn higher profits. In previous studies, the pool punishment system with the second-order punishment is shown to derive stable group cooperation^18^,^19^, but the reasons such a punishment rule is generated have not been discussed. In our study, by perceiving the punishment executer to be human, we show that such a rule could emerge spontaneously because the rule benefits the punishment executers themselves. This finding makes a significant contribution to understanding the emergence of the pool punishment system with second-order punishment.

There is low support for G-type leaders, who punish only non-contributors—the first-order free riders—, and thus, the leader does not have much capital and cannot strongly punish the first-order free riders. Thus, it is difficult to accomplish cooperation in the PGG. Intuitively, the G-type punishment is a desirable action for a group because the G-type leader focuses more on the profit of the entire group rather than his or her own. Ironically, this reduces support from followers, resulting in low cooperation in PGG. It is interesting that an L-type leader who focuses not only on profit of the group but also on his/her own profit increases the profit of the entire group as a result.

High PGG cooperation is not achieved under S-type leaders, who punish only non-supporters—the second-order free riders. This result means that S-type leaders are not beneficial for the group at all. The fear of punishment is the only incentive for followers to support the leader. The results that the support of S-type leaders is lower than that of L-type leaders reflects the antagonism of followers, which implies that the L-type punishment is a superior method that can gain and maintain support from the group. However, there is no significant profit difference between the S- and L-type leaders. Moreover, under the S-type leader, the profit difference between the followers and leader is largest, showing that the S-type leader can settle in a comparatively superior position within the group. These results show that it is reasonable for the leader in our experiment to select S-type over L-type punishment. In other words, the leader support system has a risk of tyranny by the S-type leader. This tyranny by the S-type leader should occur in reality. Acemoglu and Robinson^25^ point out the importance of governance by law and a democratic system in order to prevent leader tyranny. They use many examples of this to highlight how democratic insufficiency results in exploitation by a leader. Our experiment provides a methodology to approach this issue empirically.

We can suggest that the leader support system has the effect of suppressing retaliation or anti-social punishment by free riders, which is observed in peer punishment^26^,^27^,^28^. Under the leader support system, resources tend to be concentrated with the leader. Therefore, even if followers can retaliate against their leader, they will hesitate to do so because they predict a strong retaliation from the leader. As a result, the leader support system can easily maintain unilateral punishment. We should note, however, that unilateral punishment results in good consequences for the group only under L-type leaders. Under S-type leaders, stable unilateral punishment means stable tyranny.

Since this experiment is the first attempt to demonstrate the function of a leader support system, many questions remain for future investigation. First, the influence of different payoff structures should be tested. If punishment power were stronger, that is, the punishment rate were more than double or the initial token given to punishment were more than 20, more leaders would tend to focus only on punishment against non-supporters. This is because leaders would make less effort to induce support from their followers by punishing non-contributors and being recognized as effective leaders when punishment is powerful enough to expropriate a group. In this case, the emergence of the linkage-type leader might be limited only in a relatively lower punishment power condition. Investigating different parameters would lead to a better understanding of the critical factors for emergence of linkage-type leaders. Second, manipulating the leader punishment type should be important. In this study, the analysis of leader punishment type within the support-present condition is a post-hoc analysis and we do not manipulate the leader type directly. As we set the condition in which leaders can freely chose who to punish, we reveal that some leaders spontaneously choose the linkage type punishment but causality of leader punishment and followers’ behavior is unclear. To overcome this problem, conducting an experiment that directly manipulates a leader’s punishment would be necessary. By doing so, it would be possible to clarify how punishment type causes followers’ contributions and support. Third, it is important to explore how leader support systems emerge. In our research, the leader support system is given exogenously. In order to examine how the leader support system emerges, it is necessary to set an experimental situation in which the leader is not present in the initial state of the experiment. Specifically, three stages should be set: a PGG stage in which all members participate; a support stage in which anyone can support anyone: and a punishment stage in which anyone can punish anyone. In such an experiment, it would be possible to examine how high levels of group cooperation are achieved from concentrated support for a specific individual who spontaneously executes a linkage punishment. If no one receives any information, concentrated support for a specific individual would be less likely to occur. In real-world societies, people share their characteristics or qualities as a leader with one another and sometimes, concentration of support for a charismatic individual occurs. Even in the laboratory, governance by a specific individual might spontaneously arise when participants share the qualities of each individual. Empirically investigating the natural rise of governance could be regarded as investigating the creation of a leviathan^29^ in a laboratory. Recently, some research experimentally investigates how leviathans are built^30^,^31^. This is an ambitious aim, and our idea is an important approach to investigate this topic.

## Methods

The Waseda University Ethical Review Board specifically approved this study. The methods were carried out in accordance with the approved guidelines.

### Participants

In total, 270 university students participated in this experiment, of which 162 students (27 groups) participated in the support-present condition and 108 students (18 groups) participated in the no-support condition. Participants were recruited via a university portal website, and monetary reward was emphasized during recruitment. Written informed consent was obtained from all participants prior to beginning the experiment. For 15 other participants in the support-present condition, a 6-person group could not be assembled because there were not enough members. These groups were handled by adding as participants staff members who did not know the experiment details, and thus, these groups were excluded from the analysis.

### Procedure

Eighteen participants participated in each session of the experiment. After reading explanations of PowerPoint slides, the participants answered confirmation tests that questioned their understanding of the experiment details. Neutral words were selected for explanation. After confirming that all participants understood the experiment details, they were allocated randomly to one of three six-person groups. After running a trial period once, the participants started the real session.

The details of the experimental transactions are as follows. First, at the beginning of the session, the roles of one leader who executes punishment and five followers who engage in the PGG were selected randomly. The participants were told that these roles and the composition of the group members would remain unchanged throughout the experiment. The transactions comprised three stages: a PGG stage, a support stage, and a punishment stage. The participants were told before the beginning of the experiment that these periods would be repeated 15 times, and that the tokens they earned during transactions would be redeemed as monetary remuneration.

#### PGG stage

Each of the six members, including the leader, was given 100 tokens at the beginning of the stage. The five followers decided whether to contribute all 100 tokens to the group pool or not at all. The tokens each follower contributed were doubled and distributed equally to five followers except for the leader. This meant that each time one follower made a contribution, all five followers received 40 tokens each. The leader was completely independent from the other followers. Although the leader was given 100 tokens, like the other followers, she/he did not make decisions during this stage and simply earned 100 tokens.

#### Support stage

In the support-present condition, an additional 20 tokens were provided to each of the six members, including the leader. The five followers other than the leader decided whether to provide (support) the 20 tokens to the leader or not. If a follower decided to support the leader, the follower lost the 20 tokens and the leader obtained the 20 tokens. There was nothing for the leader to decide.

In the no-support condition, the leader was given 120 tokens while the five followers were given 20 tokens each. There was nothing for any group follower or the leader to decide in this condition. The reason why only the leader was given 120 tokens is that it is the maximum attainable amount in the support-present condition if all five followers were to provide their 20 tokens to a leader. By setting the punishable amount to at least the same as the leader in the no-support condition as in the support-present condition in the subsequent punishment stage, we ensured there was no disadvantage for the no-support condition.

#### Punishment stage

The leader used the amount earned in the support stage as capital, that is, a fixed 120 tokens in the no-support condition; 20 + (the number of support followers) × 20 in the support-present condition (minimum 20, maximum 120). Then, the leader determined, in increments of 20 tokens, how much to reduce each follower’s tokens. The punishment rate was double, meaning that if a leader used 20 tokens to punish a certain follower, the follower would lose 40 tokens. As long as there was sufficient capital, the leader could reduce anyone’s amount of tokens. The amount the leader did not use for punishment was added to the leader’s own profit.

The PGG results, that is, who contributed or did not contribute to the group, were provided to all six members after the support stage in accordance with previous pool punishment system studies^18^,^19^,^20^, in which the PGG result was unknown at the stage at which participants decided whether to bear the punishment cost to the pool system. Traulsen *et al*.^2^ argued that the nature of the pool punishment system is to decide whether to support the punishment organization to establish the organization before the results of PGG. We applied this assumption in our leader support system for compatibility with previous studies. In addition, all six members were informed about followers who supported the leader after the support stage. Thus, during the punishment stage, the leader was able to decide whom to punish after ascertaining who contributed in the PGG and who supported him/her. Furthermore, all members were informed who had been punished and by how much immediately after the leader’s decision. Therefore, followers could know the punishment type of their leader.

These three stages were repeated 15 times. Experiment control was conducted using Ztree^32^. The participants wrote down the results of each stage, such as who contributed, who supported, and who was punished, on the form they were given before the experiment, and thus, they could refer to all previous results in every decision-making stage (for further information about the procedure, see Supplementary methods). The total attained score was converted to money using the rate 1 token = 0.7 yen, and the converted amount was provided plus 500 yen (the show-up fee) given to participants who concluded the experiment. The average remuneration amount was 2,117 yen.

## Additional Information

**How to cite this article**: Ozono, H. *et al*. Solving the second-order free rider problem in a public goods game: An experiment using a leader support system. *Sci. Rep.* **6**, 38349; doi: 10.1038/srep38349 (2016).

**Publisher's note:** Springer Nature remains neutral with regard to jurisdictional claims in published maps and institutional affiliations.

## Supplementary Material

Supplementary Information

## References

[b1] Hardin G. The Tragedy of the Commons. Science 162, 1243 (1968). 5699198

[b2] Olson M. The Logic of Collective Action: Public Goods and the Theory of Groups (Harvard University Press, Cambridge, 1965).

[b3] Fehr E. & Gächter S. Cooperation and punishment in public goods experiments. Am. Econ. Rev 90, 980–994 (2000).

[b4] Fehr E. & Gächter S. Altruistic punishment in humans. Nature 415, 137–140 (2002). 11805825 10.1038/415137a

[b5] Ostrom E., Walker J. & Gardner R. Covenants with and without a sword: Self governance is possible. Am. Pol. Sci. Rev 86, 404–417 (1992).

[b6] Rand D. G., Dreber A., Ellingsen T., Fudenberg D. & Nowak M. A. Positive interactions promote public cooperation. Science 325, 1272–1275 (2009). 19729661 10.1126/science.1177418 PMC2875121

[b7] Boyd R. & Richerson P. Punishment allows the evolution of cooperation (or anything else) in sizable groups. Ethol. Sociobio 13, 171–195 (1992).

[b8] Panchanathan K. & Boyd R. Indirect reciprocity can stabilize cooperation without the second-order free rider problem. Nature 432, 499–502 (2004). 15565153 10.1038/nature02978

[b9] Cinyabuguma M., Page T. & Putterman L. Can second-order punishment deter perverse punishment? Experimental Economics, 9, 265–279 (2006).

[b10] Boyd R., Gintis H., Bowles S. & Richerson P. J. The evolution of altruistic punishment. Proc Natl Acad Sci USA 100, 3531–3535 (2003). 12631700 10.1073/pnas.0630443100 PMC152327

[b11] dos Santos M., Rankin D. J. & Wedekind C. The evolution of punishment through reputation. Proc. R. Soc. Lond. B 278, 371–77 (2011). 10.1098/rspb.2010.1275 PMC3013410 20719773

[b12] Barclay P. Reputational benefits for altruistic punishment. Evo. Hum. Beh. 27, 325–344 (2006).

[b13] Pinker S. The false allure of group selection. http,//edge.org/conversation/the-false-allure-of-group-selection (2012).

[b14] Raihani N. J. & Bshary R. The reputation of punishers. Trends. Ecol. Evol. 30, 98–103 (2015). 25577128 10.1016/j.tree.2014.12.003

[b15] Guala F. Reciprocity: Weak or strong? What punishment experiments do (and do not) demonstrate. Behav. Brain Sci. 35, 1–59 (2012). 22289303 10.1017/S0140525X11000069

[b16] Yamagishi T. The provision of a sanctioning system as a public good. J. Pers. Soc. Psychol 51, 110–116 (1986).

[b17] Yamagishi T. The provision of a sanctioning system in the United States and Japan. Social. Psycho. Quarterly 51, 265–271 (1988).

[b18] Sigmund K., De Silva H., Traulsen A. & Hauert C. Social learning promotes institutions for governing the commons. Nature 466, 861–863 (2010). 20631710 10.1038/nature09203

[b19] Perc M. Sustainable institutionalized punishment requires elimination of second-order free-riders. Scient. Rep. 2, 344 (2012). 10.1038/srep00344 PMC3315691 22468228

[b20] Traulsen A., Röhl T. & Milinski M. An economic experiment reveals that humans prefer pool punishment to maintain the commons. Proc. R. Soc. B. 279, 3716–3721 (2012). 10.1098/rspb.2012.0937 PMC3415907 22764167

[b21] Ostrom E. Governing the Commons: The Evolution of Institutions for Collective Action (Cambridge University Press, Cambridge, 1990).

[b22] Matsumoto Y. & Jin N. Co-evolution of leader traits and member traits in social dilemmas (in Japanese). Japanese J. Exp. Soc. Psycho. 50, 15–27 (2010).

[b23] Baldassarri D. & Grossman G. Centralized sanctioning and legitimate authority promote cooperation in humans. Proc. Natl. Acad. Sci. USA 108, 11023–11027 (2011). 21690401 10.1073/pnas.1105456108 PMC3131358

[b24] O’Gorman R., Henrich J. & Van Vugt M. Constraining free riding in public goods games: Designated solitary punishers can sustain human cooperation. Proc. Biol. Sci. 276, 323–329 (2009). 18812292 10.1098/rspb.2008.1082 PMC2674351

[b25] Acemoglu D. & Robinson J. Why Nations Fail: The Origins of Power, Prosperrity, and Poverty (Crown Business, New York, 2012).

[b26] Nikiforakis N. Punishment and counter-punishment in public good games: Can we really govern ourselves? J. Public Econ. 92, 91–112 (2008).

[b27] Herrmann B., Thöni C. & Gächter S. Antisocial punishment across societies. Science 319, 1362–1367 (2008). 18323447 10.1126/science.1153808

[b28] Cinyabuguma M., Page T. & Putterman L. Cooperation under the threat of expulsion in a public goods experiment. J. Pub. Econ. 89, 1421–1435 (2006).

[b29] Hobbs T. Leviathan: Or the Matter, Forme, and Power of a Common-Wealth Ecclesiasticall and Civill, ed. by Ian Shapiro (Yale University Press; 2010) (1651).

[b30] Gross J., Méder Z. Z., Okamoto-Barth S. & Riedl A. Building the Leviathan–Voluntary centralisation of punishment power sustains cooperation in humans. Scient. Rep. 6, 20767 (2016). 10.1038/srep20767 PMC4757890 26888519

[b31] Nicklisch A., Grechenig G. & Thöni C. Information-sensitive Leviathans. J. Pub. Econ. 144, 1–13 (2016).

[b32] Fischbacher U. z-Tree: Zurich toolbox for ready-made economic experiments. Experimental Economics 10, 17–178 (2007).

