# Supplementary material for: Solving the second-order free rider problem in a public goods game: An experiment using a leader support system
Source: Sci Rep. 2016 Dec 9;6:38349. doi: 10.1038/srep38349 (PMC5146942; doi:10.1038/srep38349)
Supplement: Supplementary Information [file srep38349-s1.pdf]

Supplementary Information

Solving the second-order free rider problem in a public goods game:

An experiment using a leader support system

Hiroki Ozono, Nobuhito Jin, Motoki Watabe, and Kazumi Shimizu

Supplementary Table S1

Supplementary Table S1 shows the summary of leaders' punishment and followers' behavior in each group for the 15 periods in the support-present condition. The leaders of Groups 1 to 10 are linkage punishment leaders (L-type) because they punish both non-contributors and non-supporters. The leaders of Groups 11 to 15 are self-focused punishment leaders (S-type) because they punish only non-supporters. The leaders of Groups 16 to 22 are group-focused punishment leaders (G-type) because they punish only non-contributors. The leaders of Groups 23 to 27 are other types. The leaders of Groups 23 to 25 punish only followers who neither contribute nor support leaders, and thus, it is difficult to categorize either L-, S- or G-type leaders. The leaders of Groups 26 and 27 punish followers who contribute and support leaders.

Table S1. Summary of leaders' punishment and followers' behavior in the support-present condition.

Group ID	% of punishing contributors & supporters		% of punishing contributors & non-supporters		% of punishing non-contributors & supporters		% of punishing non-contributors & non-supporters		Leader type	Average total PGG contribution	Average total support for a leader	Average profit of a leader	Average profit of followers
1	0%	(0/71)*	**100%**	(3/3)	**100%**	(1/1)	-**	(0/0)	L	493.3	96.0	208.0	196.3
2	0%	(0/68)	**100%**	(3/3)	**100%**	(1/1)	100%	(3/3)	L	473.3	92.0	198.7	190.9
3	0%	(0/62)	**22%**	(2/9)	**100%**	(1/1)	**67%**	(2/3)	L	473.3	84.0	194.7	194.1
4	0%	(0/67)	**100%**	(4/4)	**100%**	(2/2)	**50%**	(1/2)	L	473.3	92.0	176.0	181.9
5	0%	(0/66)	**100%**	(4/4)	**100%**	(3/3)	**100%**	(2/2)	L	466.7	92.0	189.3	185.9
6	0%	(0/58)	**75%**	(9/12)	**100%**	(3/3)	**100%**	(2/2)	L	466.7	81.3	158.7	180.0
7	0%	(0/64)	**100%**	(1/1)	**100%**	(7/7)	**100%**	(3/3)	L	433.3	94.7	182.7	174.9
8	0%	(0/56)	**67%**	(2/3)	**50%**	(3/6)	**80%**	(8/10)	L	393.3	82.7	160.0	165.1
9	0%	(0/47)	**50%**	(1/2)	**9%**	(1/11)	**40%**	(6/15)	L	326.7	77.3	180.0	162.9
10	0%	(0/12)	**20%**	(1/5)	**21%**	(7/34)	**42%**	(10/24)	L	113.3	61.3	156.0	120.3
11	0%	(0/22)	**40%**	(2/5)	0%	(0/36)	**100%**	(12/12)	S	180.0	77.3	172.0	130.4
12	0%	(0/10)	**38%**	(3/8)	0%	(0/43)	**43%**	(6/14)	S	120.0	70.7	168.0	120.8
13	0%	(0/13)	**80%**	(4/5)	0%	(0/42)	**60%**	(9/15)	S	120.0	73.3	165.3	118.1
14	0%	(0/8)	**75%**	(3/4)	0%	(0/44)	**74%**	(14/19)	S	80.0	69.3	160.0	110.4
15	0%	(0/2)	**50%**	(1/2)	0%	(0/26)	**24%**	(11/45)	S	26.7	37.3	138.7	110.4
16	0%	(0/11)	0%	(0/14)	**27%**	(3/11)	**15%**	(6/39)	G	166.7	29.3	132.0	140.5
17	0%	(0/12)	0%	(0/8)	**25%**	(1/4)	**16%**	(8/51)	G	133.3	21.3	129.3	137.6
18	0%	(0/11)	0%	(0/8)	**4%**	(1/25)	**23%**	(7/31)	G	126.7	48.0	153.3	129.9
19	0%	(0/7)	0%	(0/12)	**14%**	(4/28)	**21%**	(6/28)	G	126.7	46.7	153.3	130.7
20	0%	(0/4)	0%	(0/12)	**5%**	(2/41)	**78%**	(14/18)	G	106.7	60.0	158.7	120.8
21	0%	(0/8)	0%	(0/6)	**4%**	(1/28)	**9%**	(3/33)	G	93.3	48.0	161.3	126.4
22	0%	(0/3)	0%	(0/2)	**13%**	(4/31)	**44%**	(17/39)	G	33.3	45.3	134.7	105.3
23	0%	(0/5)	0%	(0/7)	0%	(0/38)	**28%**	(7/25)	Other	80.0	57.3	166.7	120.3
24	0%	(0/3)	0%	(0/4)	0%	(0/5)	**5%**	(3/63)	Other	46.7	10.7	126.7	125.6

25	0%	(0/4)	0%	(0/1)	0%	(0/51)	**68%**	(13/19)	Other	33.3	73.3	156.0	97.1
26	**20%**	(13/64)	0%	(0/1)	**100%**	(1/1)	**100%**	(9/9)	Other	433.3	86.7	156.0	169.1
27	**75%**	(3/4)	**100%**	(3/3)	**9%**	(3/32)	**33%**	(12/36)	Other	46.7	48.0	129.3	104.3

* Parentheses indicate actual numbers of punishments for each follower's type. For example, 0/71 in "% of punishing contributors & supporters" in Group 1 means that there are 71 followers who contribute to the group pool and support their leaders throughout the 15 periods in this group and the leader punishes 0 in total.
** The leader of Group 1 never encounters followers who do not contribute and do not support their leaders throughout the 15 periods, but this leader punishes both non-contributors and non-supporters. Thus, we regard the leader as L-type.

Supplementary analysis 1

Analysis including groups in which leaders punished followers who contributed and supported leaders

In the main text, we show the analysis that excludes the data of two groups (26 and 27), in which the leaders punish followers who contribute and support the leaders, because of interpretation difficulties. We describe the results of the analysis, including these two groups below.

First, we describe the comparison between L- and NL-types. We categorize the leaders of the two groups as NL-type because these two leaders behave differently from the typical L-type leaders, who punish only non-contributors and non-supporters. A Mann–Whitney U-test is conducted in this categorization and the results show the same tendencies as the results in the main text. There is a significant difference in all indexes; PGG contribution, $p < .001$, L-type higher; support for the leader, $p < .001$, L-type higher; profit of the leader, $p < .001$, L-type higher; profit of followers, $p < .001$, L-type higher.

In addition, when we ignore the punishment to followers who contribute and support their leaders, we could categorize the leader of Group 26 as NL type and the leader of Group 27 as L type. A Mann–Whitney U-test is conducted in this categorization and the results show the same tendencies as the results in the main text: there is a significant difference in all indexes; PGG contribution, $p < .001$, L-type higher; support for the leader, $p = .002$, L-type higher; profit of the leader, $p = .007$, L-type higher; profit of followers, $p = .002$, L-type higher.

Second, we perform the comparison among L-, S- and G-type leaders. When we ignore the punishment to followers who contribute and support their leaders, we can categorize the leader of Group 26 as G type and the leader of Group 27 as L type. A Mann–Whitney U-test is conducted in this categorization. Bonfirroni's correction is used to determine the significance of the comparisons of the three leader types L, S, and G from this point onward. The results show the same tendencies as the results in the main text: PGG contribution, L versus S, $p = .052$, L-type higher, L versus G, $p = .037$, L-type higher; support for the leader, L versus S, $p = .087$, L-type higher, L versus G, $p = .005$, L-type higher; profit of the leader, L versus S, $p = .693$, L versus G, $p = .022$, L-type higher; profit of followers, L versus S, $p = .055$, L-type higher, L versus G, $p = .011$, L-type higher.

In conclusion, the findings reported in the main text are robust, even when we include the data of the two groups in which the leaders punished followers who contribute and support their leaders.

Supplementary analysis 2

2.1. Analysis with the leader categorization that are less sensitive to single decisions

The categorization of leaders in the main text might have a problem because a leader's single decision shifts a leader from one type to another. For example, when a leader who punishes only non-contributors throughout the first 14 periods punishes the follower who contributes and does not support the leader only in the 15th period, this leader's type shifts from G-type to L-type due to this single decision. This sensitive categorization means the leader type can change easily and readers might question the validity of the analysis in the main text.

Here, we perform two analyses that are less sensitive to single decisions. First, we regard punishments of each follower type of less than 5% as zero. In this categorization, the leaders of Groups 18, 20, and 21, who are G-type in the main analysis, shift to the other type. A Mann–Whitney U-test is conducted in this categorization and the results show the same tendencies as the results in the main text: PGG contribution, L versus S, p= .013, L-type higher, L versus G, p = .042, L-type higher; support for the leader, L versus S, p = .024, L-type higher, L versus G, p = .003, L-type higher; profit of the leader, L versus S, p = .315, L versus G, p = .006, L-type higher; profit of followers, L versus S, p = .008, L-type higher, L versus G, p = .041, L-type higher.

Second, we regard punishments to each follower type of less than 10% as zero. In this categorization, the leaders of Groups 18, 20, and 21, who are G type in the main analysis, shift to the other type and the leader of Group 9, who is L type in the main analysis, shifts to S type. A Mann–Whitney U-test is conducted in this categorization and the results also show the same tendencies as the results in the main text: PGG contribution, L versus S, p = .012, L-type higher, L versus G, p = .059, L-type higher; support for the leader, L versus S, p = .019, L-type higher, L versus G, p = .004, L-type higher; profit of the leader, L versus S, p = .573, L versus G, p = .008, L-type higher; profit of followers, L versus S, p = .008, L-type higher, L versus G, p = .059, L-type higher.

In conclusion, the tendencies reported in the main text are strong, even in the less sensitive categorizations of leader punishment types.

2.2. Analysis with categorization of leaders by cluster analysis

We categorize leader punishment types without a priori assumptions. We perform cluster analysis with Ward's method, in which clustering variables are the percentages of punishment for each four follower types; the followers who contribute and support their leaders, who contribute and do not support their leaders, who do not contribute but support their leaders, and who do not contribute and do not support their leaders. The data of Group 1 are eliminated because this group do not have the data of punishment to followers who do not contribute and do not support their leaders. Figure S1 shows the results of the cluster analysis. A solution with three clusters is utilized in the present analyses. Only three leaders, those of Groups 8, 9, and 10, except for other types, are clustered in the different groups from the original categorization of L, S, and G types. This result indicates that these three clusters are very similar to the original categorization of L, S, and G types.

We calculate the means of the important indexes (see Table S2). Cluster 1 leaders strongly punish both non-contributors and non-supporters. The Wilcoxon matched-pairs signed-rank test reveals there is no

difference among the punishment to three follower types, that is, followers who contribute and do not support their leaders, followers who do not contribute but support their leaders, and followers who do not contribute and do not support their leaders (*p*s.>10). Therefore, Cluster 1 leaders can be regarded as L type. Cluster 2 leaders punish followers who contribute and do not support their leaders and followers who do not contribute and do not support their leaders more than those who do not contribute but support their leaders (Wilcoxon matched-pairs signed-rank test, *p*s<. 001). This means that Cluster 2 leaders focus more on punishment to non-supporters, and thus, they can be regarded as S-type leaders. Cluster 3 leaders punish followers who do not contribute but support their leaders, and those who do not contribute and do not support their leaders more than those who contribute but do not support their leaders (Wilcoxon matched-pairs signed-rank test, *p*s<. 001); thus, they can be regarded as G-type leaders.

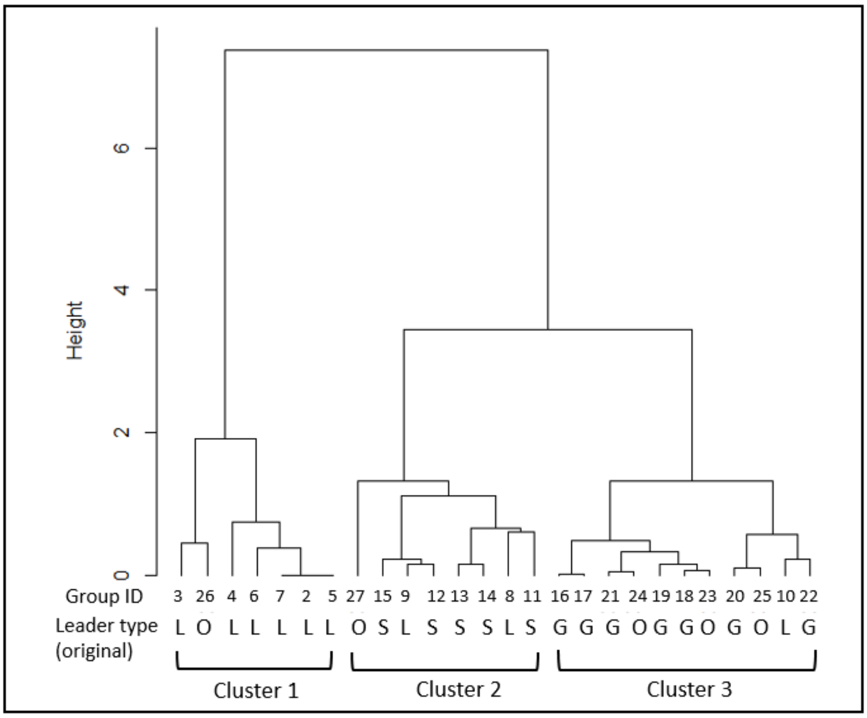

Figure S1. Cluster dendrogram of punishment behavior by leaders

A Mann–Whitney U-test is conducted in this clustering and the results show homogeneous tendencies of the results in the original categorization of L-, S-, and G-type leaders. PGG contribution, Cluster 1 versus Cluster 2, p= .009, Cluster 1 higher, Cluster 1 versus Cluster 3, $p < .001$, Cluster 1 higher; support for the leader, Cluster 1 versus Cluster 2, $p = .001$, Cluster 1 higher, Cluster 1 versus Cluster 3, $p < .001$, Cluster 1 higher; profit of the leader, Cluster 1 versus Cluster 2, $p = .263$, Cluster 1 versus Cluster 3, $p = .006$, Cluster 1 higher; profit of followers, Cluster 1 versus Cluster 2, $p = .001$, Cluster 1 higher, Cluster 1 versus Cluster 3, $p < .001$, Cluster 1 higher. We consistently find the same results in this more objective categorization, and thus, we conclude that the categorization of L-, S-, and G-type leaders in an original way is valid and reasonable.

Table S2. Comparison among groups categorized by cluster analysis

	Mean % of punishing contributors & supporters	Mean % of punishing contributors & non-supporters	Mean % of punishing non-contributors & supporters	Mean % of punishing non-contributors & non-supporters	Average total PGG contribution	Average total support for a leader	Average profit of a leader	Average profit of followers
Cluster 1 (n=7)	2.9%	71.0%	100.0%	88.1%	460.0	89.0	179.4	182.4
Cluster 2 (n=8)	9.4%	62.4%	8.6%	56.8%	161.7	67.0	159.2	127.8
Cluster 3 (n=11)	0.0%	1.8%	10.2%	31.7%	96.4	45.6	148.0	123.1

2.3. Analysis without categorization of punishment type

Here, we demonstrate the analysis without categorized punishment type, because categorized punishment types L, S, and G might be somewhat arbitrary.

In the support-present condition, cooperation levels are clearly polarized (see Figure 1). We perform cluster analysis with Ward's method, in which clustering a variable is a PGG contribution. The results are shown in Figure S2. The results reveal that groups are categorized as high cooperation groups (N=10, from 326.7 to 493.3 for average total PGG contribution) and low cooperation groups (N=17, from 26.7 to 180.0 for average total PGG contribution).

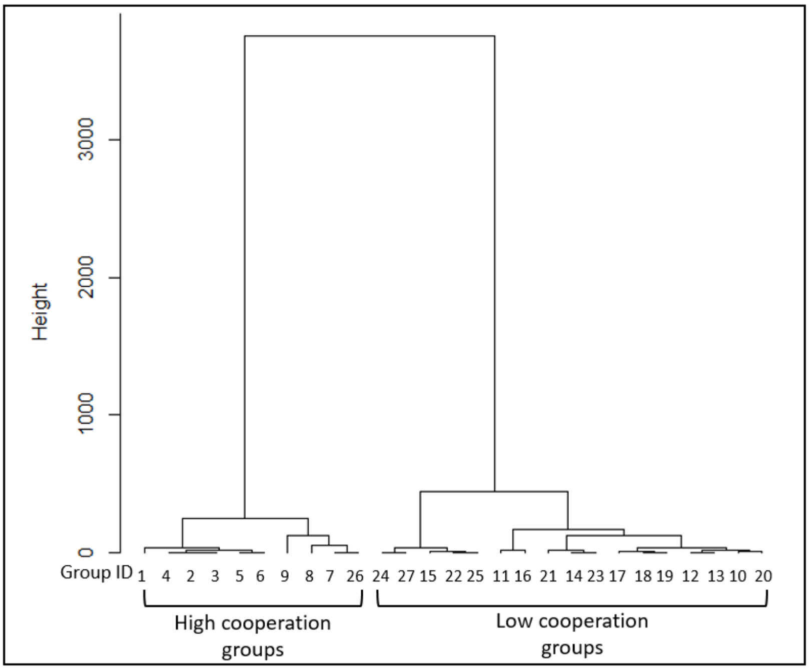

Figure S2. Cluster dendrogram of PGG contribution

We compare the punishment of leaders in high cooperation groups with those in low cooperation groups in order to investigate why this polarization occurred. Table S3 shows the comparison between low and high cooperation groups for the 15 periods. These results clearly indicate that the leaders of high cooperation groups are more likely to punish both non-contributors and non-supporters. In other words, strong linkage punishment by a leader leads to a high cooperation level in PGG. The Mann–Whitney U-test

reveals that the leaders of high cooperation groups are more likely to punish followers who do not contribute but support their leaders (p<.001), followers who contribute and do not support their leaders (p<.001), and followers who do not contribute and do not support their leaders (p<.001) than the leaders of low cooperation groups.

In addition, support for the leader and total profit of the leader are larger in high cooperation groups than in low cooperation groups (p< .001, p< .001, respectively), which indicates that strong linkage punishment induces support for the leader and benefits not only the group but also the leader himself or herself.

In summary, the analysis without punishment type of leaders suggests that linkage punishment leads to high group cooperation and is beneficial for the leader.

Table S3. Comparison between low and high cooperation groups

	Mean % of punishing contributors & supporters	Mean % of punishing contributors & non-supporters	Mean % of punishing non-contributors & supporters	Mean % of punishing non-contributors & non-supporters	Average PGG contribution	Average support for leader	Average profit of leader	Average profit of followers
High cooperation groups (n=10)	2.0%	71.4%	85.9%	81.9%	443.3	87.9	180.4	180.1
Low cooperation groups (n=17)	4.4%	23.7%	7.2%	40.2%	96.1	51.6	150.7	120.5

Supplementary method

1. <u>Instruction of the experiment</u>

After a brief verbal introduction, participants read the following instructions on the computer monitor telling them that they will take part in an experiment on decision making.

General Guidance

This is an experiment about decision making. You will be paid for participating, and the amount of money you will earn depends on the decisions that you and the other participants make. At the end of today's session you will be paid in cash for your decisions privately.

You will never be asked to reveal your identity to anyone during the course of the experiment. Your name will never be associated with any of your decisions.

At this time, you will be given 500 yens (= 5~6 dollars) for coming on time. All the money that you earn after this experiment will be yours to keep.

Earnings

In this experiment you are in a group of size 6 (you plus 5 others) and you will be asked to make a series of choices about how to allocate a set of tokens. You and the other subjects has been randomly assigned to the group, and you *will not* be able to know each other's identities. But the group members remained the same throughout the experiment.

The details of the experimental transactions are as follows. There are two different roles in the experiment. Five members named A, B, C, D and E will play the same role, but one member named Z will play a different role. Who will be assigned as Z will be selected randomly in the beginning of the experiment and these roles remained the same throughout the experiment. The experiment comprised three stages, 1st stage, 2nd stage and 3rd stage. These stages will be repeated 15 times, and the tokens you earn during transactions will be redeemed as monetary remuneration.

Now, let us explain the details of each stage.

1st stage:

Each of the six members, including Z, are given 100 tokens at the beginning of the stage. The members except for Z are asked to decide whether to contribute all 100 tokens to the group pool or not at all. The tokens each member contributed are doubled and distributed equally to five members except for Z. This means that each time one member make a contribution, all five members except for Z received 40 tokens each. Z was completely independent from the other members. Although Z are given 100 tokens, like the other members, s/he does not make decisions during this stage and simply earns 100 tokens.

Examples of choices you will make in this experiment and earnings

Example 1: Suppose that you are A, not Z. You and the other 4 members all contribute 100 tokens to a pool. You will earn:

100 (initial endowment) − 100 (the tokens you gave)

+ 0.4 × 500 (the sum of tokens 5 members gave)
= 200
Example 2: Suppose that you are B, not Z. You and the other 4 members all contribute nothing. You will earn:
100 (initial endowment) − 0 (the tokens you gave)
+ 0.4 × 0 (the sum of tokens 5 members gave)
= 100
Example 3: Suppose that you are B, not Z. You contribute nothing and all the other members contribute 100 tokens each. You will earn:
100 (initial endowment) − 0 (the tokens you gave)
+ 0.4 × 400 (the sum of tokens 5 members gave)
= 260.
Example 4: Suppose that you are Z. You do not make any decision. You will earn:
100 (initial endowment).

2nd stage (support-present condition):
An additional 20 tokens are provided to each of the six members, including Z. The five members other than Z decide whether to provide the 20 tokens for Z or not. If a member decides to provide his/her tokens for Z, s/he loses the 20 tokens and Z obtains the 20 tokens. There is nothing for Z to decide.

Examples of choices you will make in this experiment and earnings
Example 1: Suppose that you are A, not Z. You provide 20 tokens for Z. You will earn:
20 (initial endowment) − 20 (the tokens you provide)
= 0
Example 2: Suppose that you are B, not Z. You provide nothing for Z. You will earn:
20 (initial endowment) − 0 (the tokens you gave)
= 20
Example 3: Suppose that you are Z. You do not make any decision. A, B, C, D, and E provide 20, 0, 20, 20, and 0 to you, respectively. You will earn:
20 (initial endowment) + 60 (the tokens you are provided by the other members)
= 80.

2nd stage (no-support condition):
Z are given 120 tokens while the other five members are given 20 tokens each. There is nothing for any group members to decide in this stage

3rd stage:
Z can use the amount earned in the 2nd stage as capital, that is, the fixed 120 tokens [in the no-support condition] , 20 + (the number of members who provided their tokens) × 20 [in the support-present

condition]. Then, Z determines, in increments of 20 tokens, how many tokens to reduce from A to E. If Z uses 20 tokens to reduce the token of a certain member, the member will lose 40 tokens. As long as there is sufficient capital, Z can reduce anyone's amount of tokens. The amount Z does not use for reduction is added to Z's own profit.

Examples of choices you will make in this experiment and earnings

Example 1: Suppose that you are A, not Z. You obtained 200 tokens in the 1st stage and 20 tokens in the 2nd stage. Z decides to reduce 40 tokens from you. You will earn:
200 (1st-stage earning) + 20 (2nd-stage earning) – 40 (the reduction by Z)
= 180 (the total earning in the period).

Example 3: Suppose that you are Z. you obtained 100 tokens in the 1st stage and 80 tokens in the 2nd stage. You decide to use 60 tokens in total to reduce the other members' tokens. You will earn:
100 (1st-stage earning) + 80 (2nd-stage earning) – 60 (that used to reduce the other members' tokens)
= 120 (the total earning in the period).

Feedback:

All six members are informed about the results of 1st stage, that is, who contributes or does not contribute to the group, after the 2nd stage. In addition, all the members are informed about members who provide their 20 tokens for Z after the 2nd stage as well. Thus, during the 3rd stage, Z is able to decide whose tokens to reduce after ascertaining who contributed in the 1st stage and who provided their tokens for Z. Furthermore, all members are informed whose tokens were reduced and by how much immediately after Z's decision.

These three stages will be repeated 15 times. The total attained score will be converted to money using the rate 1 token＝0.7 yen, and the converted amount will be provided plus 500 yen (the show-up fee) given to you at the end of this experiment.

After this general instruction above, all participants start the experiment after filling out a confirmation test.

Confirmation Test

Before you start to make your decision, we should solve all questions on the paper. Read carefully through the provided information and write down the number of points on the paper. We will watch you solving the examples, check whether you get the right answers, and help you in case there is a problem or a question.

Before the decision-making

Good, now everybody has correctly solved the problems. We will distribute the form on which you will write down the results of each stage, such as who contributed, who provided for Z, and how many tokens Z reduced from A to E (see Figure S3). Whenever you want, you can refer to the previous results by

referring to the form. If anybody has any more questions, raise your hand now. Otherwise, let us practice how to make your decisions on your computer screens and how to write down the results on the form.

The number of this period: (   )

	1st Stage		2nd Stage		3rd Stage
ID	Contribute: Y<br>Not contribute: N	Profit of this stage	Provide for Z:Y<br>Not provide for Z:N	Profit of this stage	How many tokens did Z reduce from A to E?
A					
B					
C					
D					
E					
Z		100			

Your total profit of this period : (   )

The number of this period: (   )

	1st Stage		2nd Stage	3rd Stage
ID	Contribute: Y<br>Not contribute: N	Profit of this stage	Profit of this stage	How many tokens did Z reduce from A to E?
A			20	
B			20	
C			20	
D			20	
E			20	
Z		100	120	

Your total profit of this period : (   )

Figure S3. Form in which the participants fill out the results of each period in the support-present condition (above) and the no-support condition (below)

2. <u>Screen shots of computer displays during the experiment.</u>

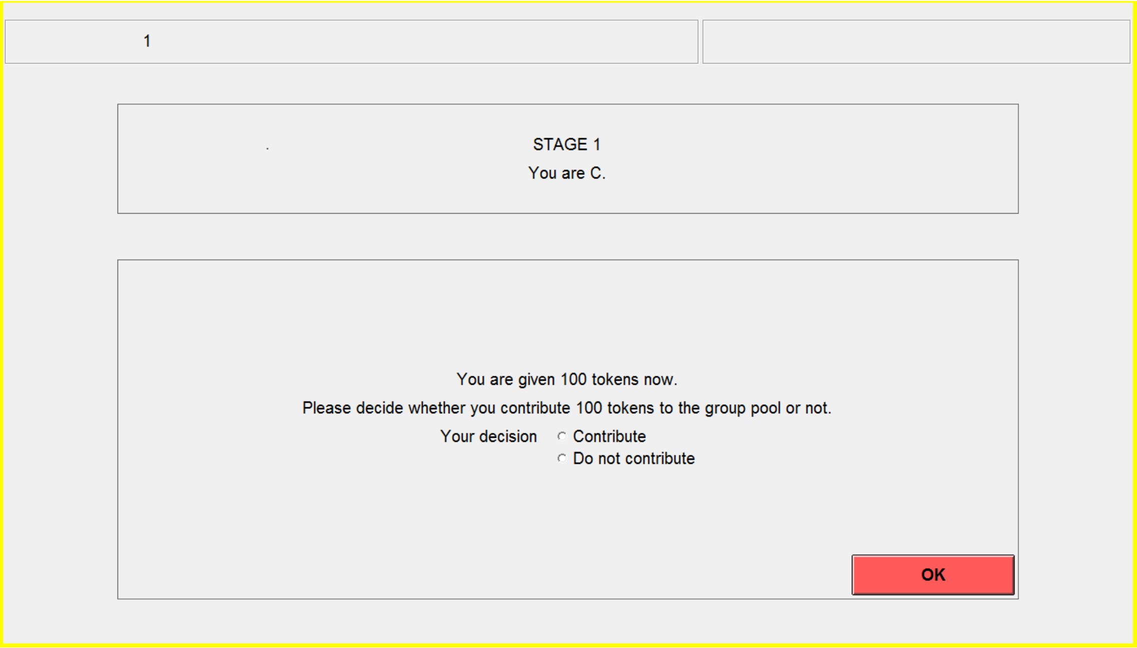

Screen shot of computer display when A, B, C, D, and E make decisions in the 1st stage.

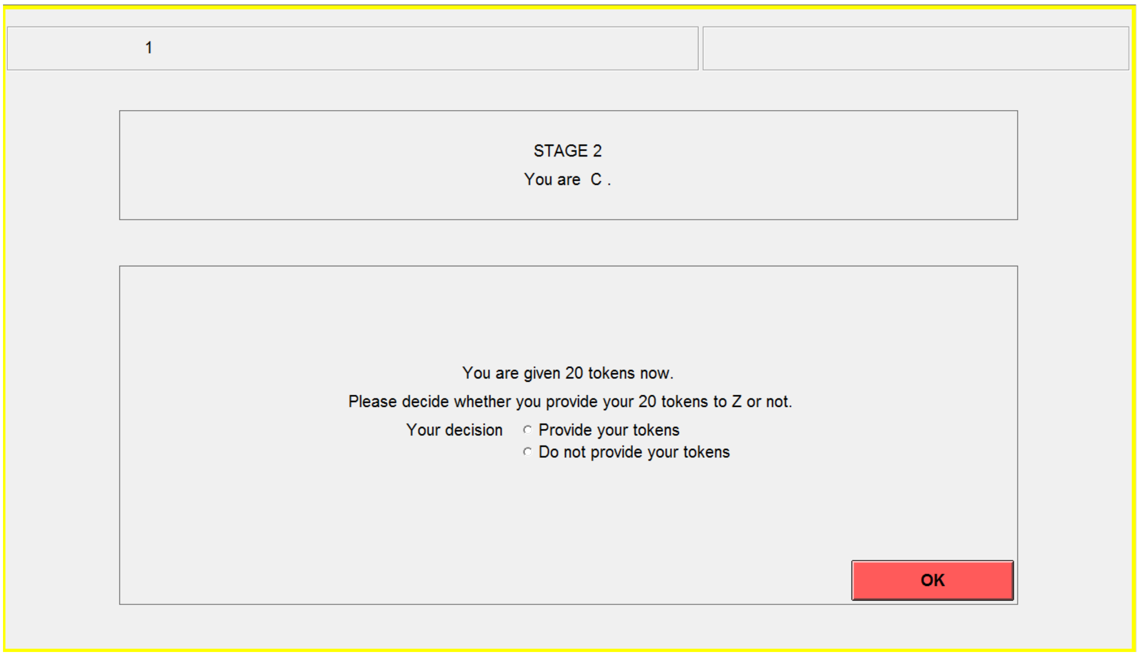

Screen shot of computer display when A, B, C, D, and E make decisions in the 2nd stage.

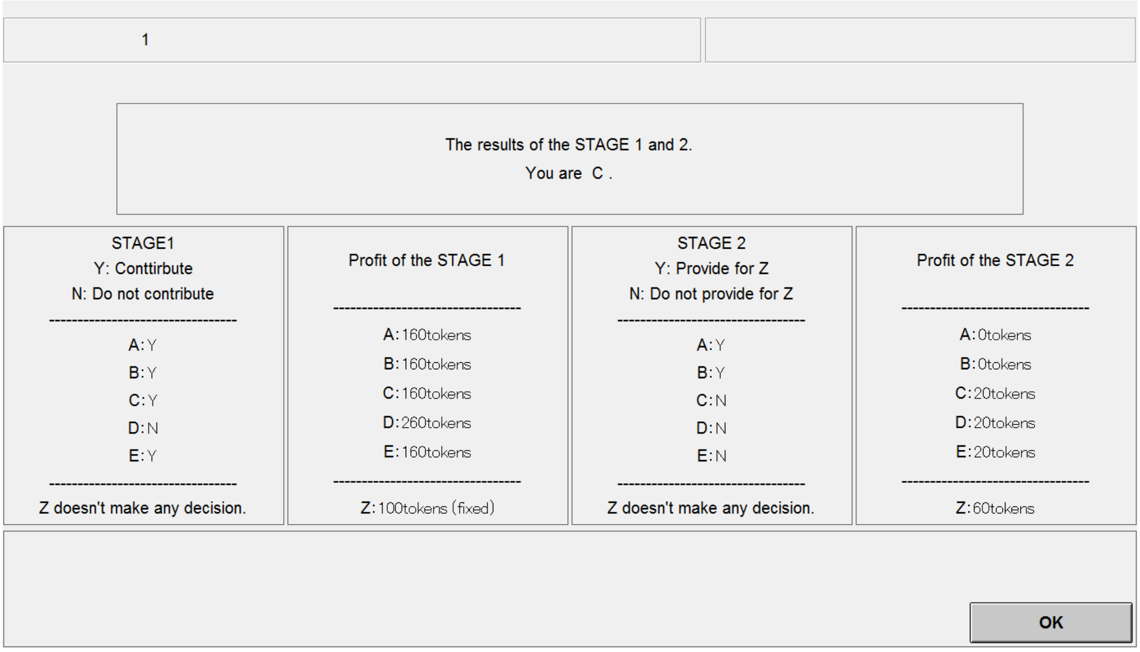

Screen shot of computer display when showing feedback after the 2nd stage.

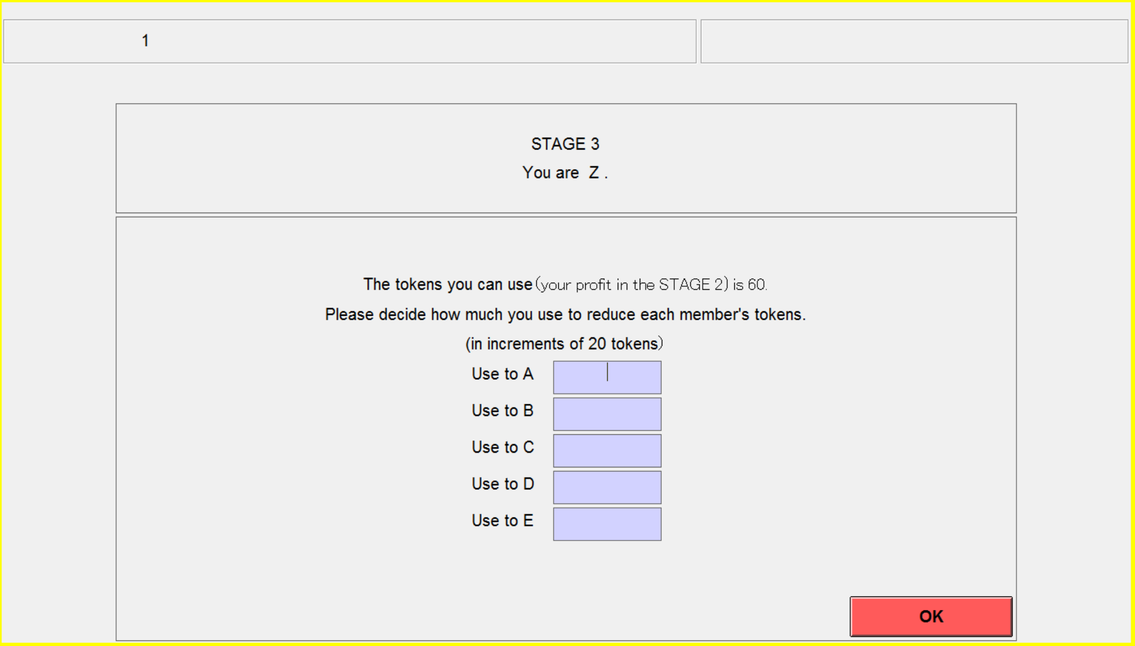

Screen shot of computer display when Z make decisions in the 3rd stage.

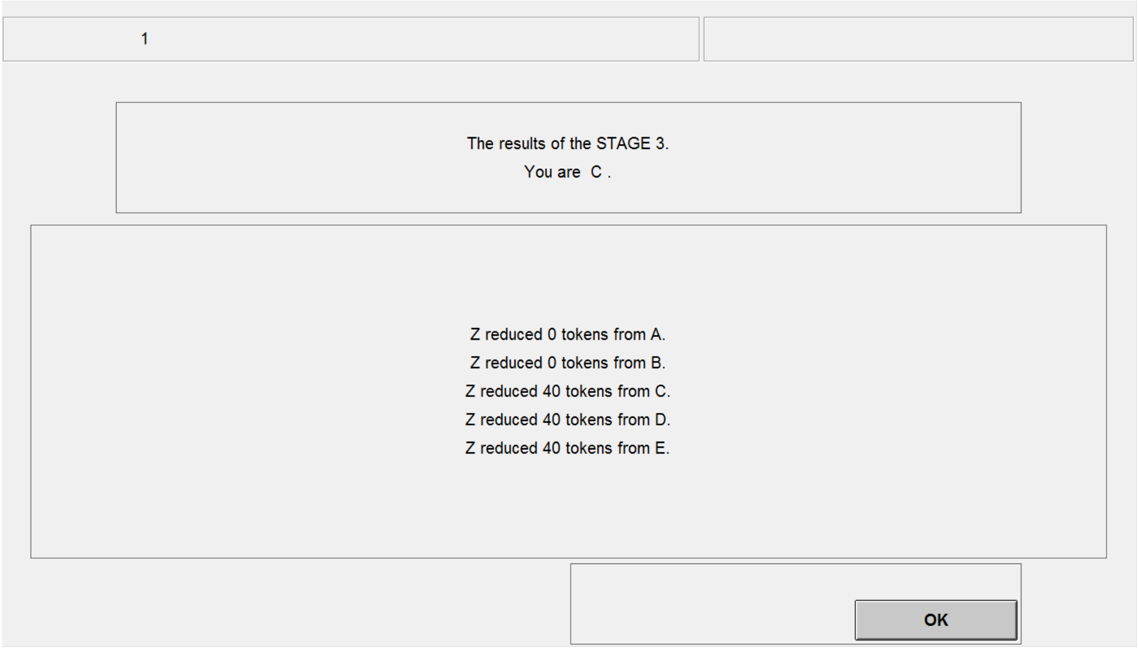

Screen shot of computer display when showing feedback after the 3rd stage.
